# Adenosine, Ketogenic Diet and Epilepsy: The Emerging Therapeutic Relationship Between Metabolism and Brain Activity

**DOI:** 10.2174/157015909789152164

**Published:** 2009-09

**Authors:** S.A Masino, M Kawamura, C.D. Wasser, L.T Pomeroy, D.N Ruskin

**Affiliations:** 1 Psychology Department, Trinity College, 300 Summit St., Hartford, CT, USA; 2 Neuroscience Program, Trinity College, 300 Summit St., Hartford, CT, USA

**Keywords:** Metabolism, neuroprotection, neurodegeneration, sleep, pain, autism, addiction, dopamine.

## Abstract

For many years the neuromodulator adenosine has been recognized as an endogenous anticonvulsant molecule and termed a “retaliatory metabolite.” As the core molecule of ATP, adenosine forms a unique link between cell energy and neuronal excitability. In parallel, a ketogenic (high-fat, low-carbohydrate) diet is a metabolic therapy that influences neuronal activity significantly, and ketogenic diets have been used successfully to treat medically-refractory epilepsy, particularly in children, for decades. To date the key neural mechanisms underlying the success of dietary therapy are unclear, hindering development of analogous pharmacological solutions. Similarly, adenosine receptor–based therapies for epilepsy and myriad other disorders remain elusive. In this review we explore the physiological regulation of adenosine as an anticonvulsant strategy and suggest a critical role for adenosine in the success of ketogenic diet therapy for epilepsy. While the current focus is on the regulation of adenosine, ketogenic metabolism and epilepsy, the therapeutic implications extend to acute and chronic neurological disorders as diverse as brain injury, inflammatory and neuropathic pain, autism and hyperdopaminergic disorders. Emerging evidence for broad clinical relevance of the metabolic regulation of adenosine will be discussed.

## INTRODUCTION

Adenosine is a potent neuromodulatory purine present throughout the extracellular space of the central nervous system. Adenosine acts at pre- and postsynaptic G protein-coupled receptor subtypes (A_1_, A_2A_, A_2B_, and A_3_) [58], and the influence of A_1_ and A_2A_ receptor subtypes predominates due to their higher affinity for adenosine (~100 nM) [100] and their activation by ongoing levels of adenosine. In brain, A_1_ receptors are distributed widely [73] and A_2A_ receptors are located preferentially in the basal ganglia and olfactory tubercle [169]. Both the A_1_ and A_2_ receptor subtypes are antagonized by caffeine, the most widely used psychoactive substance worldwide, and caffeine’s actions at adenosine A_1_ and A_2A_ receptors in the central nervous system underlie its alerting, locomotor and cognitive effects [136].

Adenosine exerts a tonic inhibition of neuronal excitability *via* A_1_ receptors in many brain regions, including hippocampus and cerebral cortex, and this baseline inhibition influences both baseline synaptic activity [132, 175] and neuronal plasticity [36]. Adenosine’s inhibitory influence also alters seizure threshold directly, and increased extracellular adenosine during a seizure plays a key role in postictal depression [193] and in keeping a seizure focus localized [140] *via* adenosine A_1_ receptors [74]. Large additional amounts of adenosine are mobilized during metabolically stressful cellular conditions such as low oxygen or glucose [57], and increased extracellular adenosine acting at the adenosine A_1_ receptor has been shown to be neuroprotective during conditions of metabolic stress [45]. Overall, adenosine holds well established and profound therapeutic potential for conditions such as stroke, brain injury, pain and epilepsy, among others [17, 79].

Whereas adenosine’s role as an endogenous neuroprotective molecule during pathology such as stroke, hypoxia and brain injury is of paramount clinical importance, and has long been the focus on adenosine-based therapies, the ongoing effects of adenosine are critical to baseline neuronal excitability and sleep behaviors. In addition, adenosine influences the risk for an epileptic seizure, and can control significantly the expression and progression of a broad range of acute and chronic neurological conditions. With an unparalleled long-term epidemiological database based on manipulating the influence of endogenous adenosine, i.e. the worldwide consumption of caffeine [60, 85], a strategy focused on regulating endogenous adenosine is likely to be well tolerated and non-toxic.

To date, receptor-based strategies to augment the inhibitory influence of adenosine by targeting A_1_ receptors have been unable to harness its clinical potential, primarily due to peripheral side effects [43, 51]. Accordingly, interest has intensified in the regulation of adenosine directly by physiological stimulation [42, 45, 76, 77], metabolism [125] and adenosine kinase [75], an astrocytic intracellular enzyme that, together with equilibrative adenosine transport, controls extracellular adenosine levels. We now appreciate the active role of astrocytes in regulating extracellular adenosine [156], underscoring the multi-faceted and direct impact that glia have on neuronal activity and signaling. Together, these recent findings highlight the dynamic regulation of adenosine by cellular and metabolic stimuli, and thus expose new clinically-relevant strategies for augmenting adenosine.

## ADENOSINE: A KEY LINK BETWEEN METABOLISM AND NEURONAL SIGNALING

As both the core of ATP and a prevalent neuromodulator, adenosine is poised to link changes in cell metabolism with changes in neuronal activity [109]. Indeed, adenosine levels rise dramatically in the extracellular space during all types of metabolic stress and earned adenosine the apt title of “retaliatory metabolite” [138] - its profound inhibitory influence at both pre- and postsynaptic receptors serves to limit energy demand and excitotoxicity when energy availability is compromised [59]. The direct release of adenosine *via* nucleoside transporters can increase extracellular adenosine under physiologically stressful conditions [114], and typically adenosine’s role as a retaliatory metabolite is thought to be mobilized when intracellular ATP dephosphorylation outstrips ATP production [145, 170]. However, the regulation of adenosine by ongoing physiological stimuli and under non-pathological conditions of adequate or even high intracellular ATP is becoming more appreciated [45, 96, 128]. In addition, degradation of extracellular ATP is a major source of extracellular adenosine [29, 44, 82], so manipulations that increase extracellular ATP have a net effect on neuromodulation by adenosine [44].

Ketogenic strategies such as fasting or adhering to a ketogenic (high-fat, low-carbohydrate) diet increase ATP and other energy molecules in brain [20, 37, 126, 134]. These metabolic manipulations are known to reduce seizures significantly [194], and have been shown to offer neuroprotection in animal models of brain injury [70, 125]. Emerging evidence suggests that mimicking key cellular aspects of ketogenic metabolism increases extracellular adenosine [96], and furthermore, that an increased influence of adenosine at the A_1_ subtype plays a key role in the anticonvulsant success of ketogenic strategies [127] *via* its combined presynaptic inhibition of glutamatergic terminals and its postsynaptic hyperpolarization *via* K^+^ channels. Due to the functional coupling and inverse relationship between adenosine and dopamine receptors (A_2A_/D_2_ and A_1_/D_1_) [66], a general increase in extracellular adenosine could also decrease dopaminergic transmission. A diverse set of physiological and pathological stimuli that modulate extracellular adenosine are outlined in Table **1**.

## ADENOSINE, METABOLISM AND EPILEPSY

The inhibitory adenosine A_1_ receptor is functionally dominant in hippocampus and cerebral cortex, and underlies adenosine’s role as an endogenous anticonvulsant [43]. Accordingly, any manipulation which increases extracellular adenosine offers significant potential for both preventing and halting epileptic seizures, the vast majority of which initiate and propagate in these forebrain regions. Unlike the point-to-point and activity-dependent changes in synaptic transmission effected by classical neurotransmitters, adenosine exerts a tonic modulatory influence on neuronal activity. Thus the anticonvulsant effects of adenosine are dissimilar mechanistically to classical actions of glutamate and GABA, the neurotransmitters targeted most often for the treatment of epilepsy [168]. Adenosine itself is not packaged in vesicles, and its non-synaptic release, such as from astrocytes and through nucleoside transporters, can influence a neural “neighborhood.” Akin to a broad-based influence of altered metabolism, such as *via* ketogenic strategies, the presence of a tonic level of adenosine in the extracellular space makes adenosine a major player in the dynamics of nervous system activity and even in determining the set point of normal physiology versus pathology in brain function – such as by determining seizure threshold. Indeed, local release of adenosine itself [89] or regulating local metabolism of adenosine by reducing its intracellular rephosphorylation by adenosine kinase [18] both offer significant emerging potential for treating epilepsy. Importantly, adenosine is effective in stopping seizures which are pharmacoresistant [16], thus opening new therapeutic opportunities for intractable epilepsy.

Physiological conditions which offer both high ATP levels and increased extracellular adenosine are particularly ideal for epilepsy and for many acute and chronic neurological disorders characterized by metabolic dysfunction and neuronal vulnerability or frank progressive neurodegeneration. Enhanced ATP levels provide energy reserves for a neuron to continue functioning under stress, essential for maintaining cell calcium and other ion gradients across the cell and mitochondrial membrane. Increased extracellular adenosine, at levels permitting normal synaptic transmission, plasticity and cognition, offers a neuroprotective buffer against insults, reduces excitation, and averts excessive ATP demand, expressly critical in cells with compromised energy capacity. Ideally, metabolic strategies enhance ATP, increase extracellular adenosine, and boost on-demand adenosine within a neural neighborhood to provide local seizure control and neuroprotection. As such, metabolic and dietary strategies such as ketogenic diet therapy may increase regional or global adenosine and increase overall seizure threshold as described below.

## THE REGULATION OF ADENOSINE AND BIOENERGETICS BY KETOGENIC METABOLISM

Multiple lines of evidence suggest that adenosine, ATP, and general cellular energy are upregulated by ketogenic metabolism. To highlight these effects, it is necessary to review the underlying biochemistry of ketosis. Due to restricted carbohydrate intake, blood glucose is very low during chronic consumption of a ketogenic diet (or prolonged fasting). During conditions of limited glucose the liver maintains energy homeostasis by converting fatty acids and some amino acids to ketone bodies (β-hydroxybutyrate, acetoacetate, acetone) which are then transported by the blood to other tissues for use as fuel. The brain is particularly dependent on this process, since it is poor both at using fatty acids directly for fuel and at converting fatty acids to ketone bodies; the brain is a unique tissue in this regard [65, 147]. While the enzymes involved in ketone body synthesis are detectable in brain, they are present at far lower levels than in liver [4, 27, 28].

Ketone bodies lead to energy production by conversion to acetyl-CoA which then enters the mitochondrial tricarboxylic acid cycle, replacing pyruvate (derived from glycolysis) as an acetyl-CoA source. The tricarboxylic acid cycle then leads as usual to proton flow out of the mitochondria matrix; this gradient in turn powers ATP production by ATP synthase in the mitochondrial inner membrane. Not only can ketone bodies substitute for glucose, metabolism of ketone bodies is more efficient than that of glucose, leading to more available energy for ATP synthesis. This effect derives from the higher heat of combustion of ketone bodies compared to pyruvate [187]; ketone body metabolism leads to reduction of the mitochondrial NAD couple (NAD^+^/NADH) and oxidation of the mitochondrial co-enzyme Q couple (Q/QH_2_). The difference between the redox potentials of these couples determines the magnitude of the proton gradient which in turn determines the free energy (ΔG’) of ATP hydrolysis – the increased difference with ketone body metabolism leads to increased ΔG’ for ATP production [187]. Key aspects of this energy cycle and its relationship to adenosine are summarized in Fig. (**1**).

Experimental application of ketone bodies *in vitro* leads to increased ATP/ADP and phosphocreatine/creatine ratios and increased levels of the tricarboxylic acid cycle substrates citrate and isocitrate [95, 173]. Overall, studies of ketogenic metabolism in the brain demonstrate an increased energetic status using measures ranging from respiration to mitochondrial density to levels of energy-storing substances such as ATP and phosphocreatine. Importantly, the brain’s bioenergetic response to ketosis appears to differ from peripheral tissues so far examined – increases in cell energy predominate in brain (Table **2**).

In addition to published neurochemical and biochemical evidence, recent neurochemical evidence suggests a regional upregulation of energy molecules in rats after maintenance on a ketogenic diet. Preliminary results demonstrate increased ATP or adenosine in specific brain regions [127, 143], including a significant increase in ATP and a strong trend toward increased adenosine in cerebral cortex [127], and upregulated ATP synthase gene expression in hippocampus [20, 141]. Field recordings from the CA1 region of hippocampal slices show a reduced electrophysiological response to hypoxia [20] and exogenous adenosine [127] after ketogenic diet therapy, consistent with increased endogenous adenosine. Hippocampal CA3 pyramidal neurons autoregulate their activity based on acute changes in intracellular ATP and extracellular glucose consistent with the metabolic consequences of ketogenic diet therapy [96]; detailed cellular mechanisms are described below.

As noted above, ketogenic metabolism occurs during restricted glucose and is characterized by increased ATP. During single-cell patch clamp recordings, when extracellular glucose is reduced but not eliminated, and the level of intracellular ATP is sufficient or high in the patch pipette, CA3 pyramidal neurons regulate their own excitability by releasing ATP, activating adenosine A_1_ receptors and opening postsynaptic K^+^ channels [96]. In contrast, during single cell recordings of CA3 pyramidal neurons, when extracellular glucose is reduced and intracellular ATP is low, CA3 pyramidal cells depolarize significantly [96]. These low ATP/low glucose conditions are similar to an ischemic stroke, where both oxidative phosphorylation and glucose availability are interrupted. Thus, an autocrine regulation of CA3 neuron excitability during low glucose depends critically on intracellular ATP; the subsequently increased extracellular adenosine likely influences nearby neurons, particularly important in CA3, a region with recurring excitatory collaterals. Overall this concurrent intracellular ATP / extracellular glucose manipulation mimicking metabolic aspects of ketogenic diet therapy provides a clear example of a situation where intracellular ATP is high yet extracellular adenosine increases – ideal for autocrine regulation by increasing seizure threshold and promoting neuronal survival.

Evidence for autocrine neuronal regulation is consistent with adenosine’s role as a neuroprotective agent, mobilized proactively under these conditions by non-pathological changes in altered metabolism. Similarly, ketone-based metabolism [35] or a ketogenic diet [70] has already shown neuroprotective properties in injury models in multiple brain regions. While it seems counterintuitive for brain cells to release ATP when extracellular glucose is low, ultimately this process can save cell energy: under conditions of sufficient ATP, there is a large ratio of ATP:adenosine (10,000:1) inside the cell. Upon releasing a relatively small amount of ATP, a coherent set of mechanisms degrade extracellular ATP to adenosine, activate adenosine A_1_ receptors and reduce excitability. Thus, releasing a relatively small amount of ATP during low glucose is a powerful preemptive strike against a condition of increased excitability – potentially placing energy demands which could exceed energy supply and compromise neuronal function.

## CLINICAL IMPLICATIONS

The clinical efficacy of a ketogenic diet is well-established for pediatric epilepsy. It has been validated retrospectively by multiple clinical sites [38, 62, 153, 188] and confirmed recently in a randomized controlled trial [135]. Similar to adenosine, the ketogenic diet is effective in medically-refractory epilepsy, and thus reduces seizures *via* mechanisms other than those targeted by antiepileptic drugs available currently [168]. A significant subset of pediatric epilepsy patients become and remain seizure-free, and can reduce or eliminate their medication even after discontinuing diet therapy. Most common side effects are short term, and include hunger, constipation, and lethargy [135, 188]. However, kidney stones can develop in 5-7% of children on the diet [171].

The ketogenic diet may also offer benefits for adult epilepsy [15], and recently has been shown to improve dramatically or even reverse the metabolic syndrome which characterizes type II diabetes [198]. Notably, a ketogenic diet was more effective than a calorically restricted diet, and virtually reversed metabolic syndrome in all persons who were able to comply with the restrictions of dietary therapy [192]. Basic research has also shown dramatic results in slowing tumor growth in brain cancer [177], and synergistic effects of the simultaneous use of a ketogenic diet and 2-deoxyglucose [121], a metabolic manipulation which inhibits glycolysis, increases adenosine [200] and decrease seizures [69, 181]. Thus, the therapeutic applications of a ketogenic diet and metabolism-based therapies are broadening rapidly beyond pediatric epilepsy to other chronic and prevalent conditions.

As a whole, the capacity for a ketogenic diet to increase specifically the influence of endogenous adenosine offers testable and clinically-relevant predictions; many of these predictions are validated by published clinical and basic research, lending further conceptual support [125, 126]. Below we highlight briefly some of the central nervous system implications of a relationship between a ketogenic diet and adenosine. A subset is supported strongly by published research, in particular neuroprotection and sleep. Data are emerging for neurodegeneration, hyperdopaminergic disorders and autism. In addition, we speculate upon major clinical implications for pain.

### Brain Injury

The attractive features of adenosine as an endogenous anticonvulsant are recapitulated in its role as a neuroprotective molecule. Increasing adenosine’s inhibitory influence *via* the adenosine A_1_ receptor protects neurons in virtually any model of brain injury [45], and the adenosine A_1_ receptor has long been a major focus of and goal for adenosine-based neuroprotective therapies. Increasing the influence of adenosine at A_1_ receptors offers neuroprotection either before [90] or after [190] the injury. Unfortunately, and akin to epilepsy, the peripheral side effects of receptor-based strategies have stymied drug development and adenosine A_1_ receptors have lost momentum as a primary neuroprotective target. 

Importantly, and as predicted based on a relationship between ketone metabolism and adenosine, the ketogenic diet or analogous metabolic strategies have shown neuroprotective properties in multiple brain injury models and brain regions, including spinal cord injury [160]. Some of these effects have been counterintuitive – neuroprotection was observed after a ketogenic strategy such as fasting [35], or after strategies that interrupted metabolism [102] or inhibited glycolysis [111]. Importantly, neuroprotection was observed even if altered metabolism was initiated after the injury [152], making this a particularly promising strategy. Based on differential consequences and responses in the central nervous system versus the periphery (Table **2**), a metabolic strategy could lend new therapies and recommendations for the treatment of brain injuries, and open a new and exciting chapter for adenosine and neuroprotection.

### Neurodegeneration

The neuroprotective effects of adenosine *via* altered metabolism may extend to neurodegenerative diseases. Metabolic dysregulation at the cellular level is a hallmark of many chronic and neurodegenerative disorders [7], and often it is unclear if it causes and/or results from the progressive neuronal dysfunction. The brain has an extremely high fat content, and as noted in Table **2** offers some differential responses to metabolic manipulations as compared to peripheral tissues. These dual and unique features of brain composition and brain metabolism enhance therapeutic opportunities for chronic and progressive disorders [1], and suggest the potential for limited or reduced peripheral side effects.

As one example, metabolic therapy may offer symptomatic delay or improvement even in a genetic, neurodegenerative disorder such as Huntington’s disease [41, 186]. Drug treatments targeting type II diabetes, which, similar to a ketogenic diet, offer improved glycemic control, can reduce symptoms and increase survival in mouse models of Huntington’s disease [116, 122]. With gene therapy still beyond the near-term horizon, metabolic treatment strategies which delay or reduce symptoms of disease could offer significant improvements in quality of life for current patients with Huntington’s disease and a host of neurodegenerative conditions. In addition, at least some neurodegenerative diseases are associated with an increased risk of seizures, such as Alzheimer’s disease [3]. It is unknown whether subclinical seizures may occur and contribute to the progression of Alzheimer’s disease or other neurodegenerative diseases in humans. Ultimately, similar to epilepsy, adenosine-based metabolic therapies could limit acute dysfunction and offer ongoing neuroprotection against progressive neuronal decline and degeneration.

### Sleep

Adenosine promotes sleep [165, 189], and treating sleep disorders and enhancing the quality of sleep is a major concern for public health [23]. The specific adenosine-based mechanism established initially as sleep-promoting was increased activity at the A_1_ receptor in the basal forebrain [166]. More recently the adenosine A_2A_ receptor has been recognized for an important role in sleep [88, 174]. The alerting effects of caffeine are attributed to its actions at adenosine receptors in forebrain areas important for cognition as well as arousal centers, and it may be that endogenous adenosine acting at both of the receptor subtypes in brain that are antagonized by caffeine – A_1_ and A_2A_ adenosine receptors – play a role in sleep behaviors [8].

In keeping with adenosine’s role as a sleep-promoter, and the potential for a ketogenic diet to increase ATP and extracellular adenosine, the ketogenic diet has been shown to improve sleep quality and quantity in children with epilepsy, especially normalizing the ultradian cycling between slow-wave and paradoxical sleep [83]. Sleep electroencephalograph (EEG) changes were observed consistently with ketogenic diet therapy, and normalization or improvement in sleep EEG was correlated with an improvement in seizures [153]. In control subjects without a diagnosis of epilepsy, effects are typically moderate and include reports of an increase in the latency to enter rapid eye movement (REM) sleep [106] and an increase in slow-wave sleep [2]. Whereas a high-fat, low-carbohydrate ketogenic diet increased slow-wave sleep, a high-carbohydrate, low-fat isocaloric diet decreased slow-wave sleep [159].

Recent evidence implicates the role of extracellular astrocyte-derived ATP as critical for the sleep-promoting influence of adenosine [82]. Furthermore, there is a well established association between poor sleep and epilepsy [105]. With such a high prevalence and cost associated with these co-morbidities, metabolic therapy appears to offer significant promise. More research is needed in this area to determine the therapeutic relationship among adenosine, a ketogenic diet and sleep.

### Autism

Multiple lines of evidence suggest that the influence of adenosine may be insufficient in autism spectrum disorders (ASD), and that increasing adenosine would reduce both physiological and behavioral hallmarks of ASD. Preliminary studies have shown that the ketogenic diet improves symptoms of autism [53], and Rett syndrome [80]. To our knowledge, dysregulation of adenosine in brain has not been tested directly with respect to symptoms of autism, although these studies are underway. Nevertheless, adenosine is a purine molecule with roles in both metabolism and neuronal signaling, and abnormalities in purine metabolism have been documented [19, 120, 142, 149, 150] in a subset of ASD.

Along with dysregulation of purine metabolism, ASD are characterized by several behavioral and physiological hallmarks of disordered adenosine: sleep disruption [25, 72, 184], markedly increased incidence of seizures [118, 195], immune dysfunction [87, 91], and reports - including self-reports - of increased anxiety and “sensitivity” and “hyperactivity” of the nervous system [24, 78, 178]. Often there is a dual diagnosis, with psychiatric comorbidities present in least 70% of individuals diagnosed with ASD. The most common comorbidities are social anxiety disorder, attention-deficit/hyperactivity disorder, and oppositional defiant disorder [178]. 

Many of the behaviors exhibited by persons with autism and/or reported by high-functioning persons with autism involve stimuli that would be predicted to release ATP and/or adenosine based on associated mechanical pressure, increased neuronal activity, decreased pH or increased temperature (ref. [45] and Table **1**). For example, rocking, spinning and Grandin’s well-known “Hug Machine” [49] all exert mechanical pressure or sudden changes in acceleration, intense intellectual activity and focus can reduce anxiety associated with ASD [78], and intense neuronal activity increases extracellular adenosine [114]. Intense exercise causes a metabolic decrease in pH [84], decreased pH has been shown to increase adenosine [42, 146], and intense exercise has been shown to increase brain adenosine [47] and improve symptoms of autism [98]. Reports show improved behavior, language and social function associated with a fever in persons with autism [30], and basic research demonstrates that a similar temperature increase in hippocampal slices increases extracellular adenosine and inhibits neuronal activity significantly [124].

Certainly many symptoms and behaviors of ASD are due to aberrant neurochemistry, neuroanatomical development and genetically-determined substrates. However, increasing the inhibitory influence of adenosine could help significantly with multiple behavioral and physiological sequelae. Importantly, increasing adenosine improves sleep, decreases seizures and reduces anxiety – all physiological effects that could be achieved by a metabolic strategy such as a ketogenic diet and would improve quality of life significantly in persons and families affected by ASD. Ketogenic diet therapy and/or increasing adenosine could improve social and behavioral symptoms and reduce or alleviate serious chronic physiological symptoms which impact the ability to learn and remember (sleep disruption/inadequate REM sleep/seizures [86, 118]) and ultimately cause permanent brain dysfunction and cognitive impairment (recurrent seizures) [25, 26]. Given adenosine’s profound effects on neuronal activity, sleep and seizures, the relationship among metabolism, autism and adenosine, including the efficacy of a ketogenic diet in reducing symptoms of autism, needs to be explored directly.

### Hyperdopaminergic Disorders

Dopamine and adenosine have long been known to be opposing at numerous levels. Behaviorally, whereas adenosine receptor antagonists (e.g. caffeine) are stimulants, such properties belong to agonists of the dopamine system (e.g. amphetamine). Biochemically, the two high affinity adenosine receptors in brain each have an antagonistic dopaminergic counterpart. Specifically, A_1_ and D_1_ receptors, and A_2A_ and D_2_ receptors, have opposing effects on 2^nd^ messenger pathways, most notably the production of cyclic AMP through heterotrimeric G-protein regulation of adenylate cyclase [61, 97]. More recently, it has become apparent that such interactions can involve direct receptor/receptor cross-talk *via* A_1_/D_1_ and A_2A_/D_2_ receptor heteromers [54]. Therefore, it is logical to explore modulation of adenosinergic activity as a possible treatment for disorders of dopaminergic function. 

Thus far there is compelling evidence that adenosine antagonists are useful in treating a hypodopaminergic disease, namely Parkinson’s disease [197]. Conversely, the ketogenic diet and an associated augmentation of adenosine may be of use in hyperdopaminergic states. The list of clinical conditions that are hypothesized to involve overactivity in the dopamine system is impressive, and includes conditions as diverse as schizophrenia, tardive dyskinesia, attention deficit/hyperactivity disorder, Tourette’s syndrome, Huntington’s disease, and drug addiction/relapse. Research in animal models of some of these disorders suggests the beneficial effects of promoting adenosine [12, 14, 39, 71, 92, 101, 113, 191]. Adenosine augmentation via ketogenic diet or analogous metabolic strategies may be particularly useful in those hyperdopaminergic disorders involving neurodegeneration, offering the combined benefits of treating symptoms as well as retarding the underlying degeneration.

Preliminary human data exist on the benefits of ketogenic diet therapy and schizophrenia [148]. Ketogenic diet therapy was tried because the physiological effects of other treatments – such as electroconvulsive shock – result in decreased blood pH, and ketone metabolism increases acid production. In addition, physicians noted carbohydrate cravings and increased intake prior to a relapse and hypothesized that persons with schizophrenia may have problematic or altered carbohydrate metabolism. Despite the small sample size and incomplete control over dietary therapy, promising results were noted [148].

Like epilepsy, schizophrenia is a chronic condition characterized by recurring episodes as well as significant proportion of cases that remain intractable to therapies available currently [107]. Finally, given the high coincident rates of epilepsy and schizophrenia [164] and type II diabetes and schizophrenia [39, 139, 144, 179], and the success of a variety of low carbohydrate and ketogenic diets in treating type II diabetes [198] and adult epilepsy [5, 103, 104], dietary therapy would seem like an optimal primary or adjuvant therapy to reduce all of these medical conditions.

### Pain

Control of chronic pain remains a major clinical problem. For example, the strongest analgesics, the opiates, have been viewed historically as poorly effective against neuropathic pain; only a subset of patients respond well to opiates [163]. Anticonvulsant drugs are a non non-opiate alternative used with success against neuropathic pain, yet they have their own set of undesirable side effects. Pain relief using anticonvulsants demonstrates that an overall inhibition of neuronal activity is a strategy that can alleviate pain, and so it is not surprising that adenosine persists as a prized, but problematic, target for pain relief [180]. Adenosine A_1_ receptor agonists given systemically reduce chronic pain effectively in experimental animals [31, 112], and conversely, genetic knockout of the A_1_ receptor enhances pain sensitivity [90, 196]. Clinically, systemic adenosine alleviates neuropathic pain [10], yet the presence of adenosine A_1_ receptors in the heart and other peripheral tissues, and the short biological half-life of adenosine in the blood, have stymied this type of therapy. Side effects can be avoided with intrathecal adenosine [9, 50], but this route of administration is obviously invasive.

Based on evidence that ketogenic strategies increase adenosine in the central nervous system, they will also be likely to alleviate pain, although research on this topic has only begun [203]. The parallels between ketogenic diets and adenosine A_1_ receptor activation include their efficacy in pharmocoresistant epilepsy, and neuronal inhibition *via* anticonvulsants appears to be one mechanism for alleviating at least a subset of neuropathic pain. We suggest that adenosine-mediated central nervous system inhibition *via* metabolic strategies such as ketogenic diet therapy will be effective against neuropathic pain. 

Beyond these possible effects on the neural substrates of pain generally, a ketogenic metabolism may have beneficial effects with inflammatory pain by reducing inflammation itself through several mechanisms. 1) adenosine A_1_ receptors have been shown to be anti-inflammatory in a number of tissues, including brain [68, 110, 183, 185]. 2) Compared to glycolytic metabolism, ketolytic metabolism produces fewer reactive oxygen species, which are known to contribute to inflammation [81, 117, 187]; 3) the long-chain polyunsaturated fatty acids in the ketogenic diet activate peroxisome proliferator-activated receptors, an effect which will reduce inflammation by inhibiting nuclear factor κB and other pro-inflammatory pathways [13, 32, 33, 115]. Altogether, multiple consequences of ketone metabolism, including increased adenosine, appear to have much clinical promise as safe, effective, non-addictive treatments for chronic neuropathic and inflammatory pain conditions.

## SUMMARY

Herein we highlight major implications of the emerging relationship among adenosine, a ketogenic diet and epilepsy and provide a broad and provocative overview of a subset of the therapeutic predictions of this metabolic relationship. The clinical implications discussed in detail are supported by preliminary or historical data, or by infrequent publication in disparate fields of research, and deserve further examination and integration. An evidence–based metabolic treatment as an adjuvant or alternate strategy is particularly attractive and important as we seek cost-effective solutions for the diverse conditions highlighted herein. Together these conditions exact an enormous toll on health care and quality of life as they are chronic, prevalent, increasing (particularly diabetes and autism), and often comorbid (sleep disorders and epilepsy, for example).

In the central nervous system, adenosine offers unparalleled yet untapped opportunities for seizure protection, neuroprotection, sleep, and pain relief, among others. Understanding the regulation of adenosine helps achieve these long-standing clinical goals and extends beyond them to alleviating both acute and chronic conditions in adults and children. In summary, the metabolic relationship among adenosine, a ketogenic diet, and epilepsy could open major new therapeutic applications and avoid peripheral side effects in a way that has eluded receptor-based strategies. 

## Figures and Tables

**Fig. (1):**
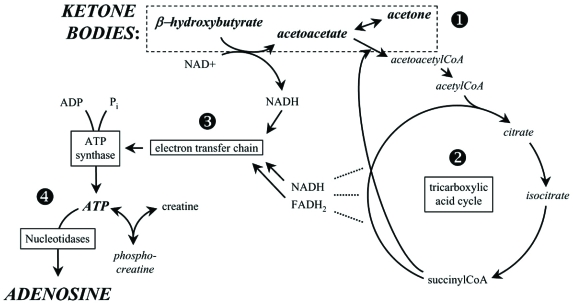
The metabolic relationship between ketones and adenosine. Compounds upregulated by a ketogenic diet or exogenous ketones are italicized. (1) During ketolytic metabolism, the ketone bodies β-hydroxybutyrate (and its breakdown products acetone and acetoacetate) are either generated locally or hepatically and transported *via* the blood to other tissues (such as brain). Ketones are converted intracellularly into acetyl-CoA which enters the tricarboxylic acid cycle. (2) This mitochondrial energy cycle generates, at multiple steps (----), protons and electrons that are channeled to the electron transport chain by NADH and FADH2 (β-hydroxybutyrate conversion to acetoacetate also contributes). Many steps of the tricarboxylic acid cycle are omitted for simplicity. (3) The electron transport chain drives an electrochemical gradient across the mitochondrial outer membrane and ultimately oxidative phosphorylation which forms ATP from ADP and phosphate (P_i_) by ATP synthase. (4) Enhanced ATP can be converted to phosphocreatine for energy storage, or broken down to its core molecule, adenosine. Adenosine levels inside and outside of the cell membrane are influenced concurrently by an equilibrative transporter. Due to the very large ATP / adenosine ratio inside the cell, a small increase in intracellular ATP could translate into a large relative increase in intracellular, and thus extracellular, adenosine.

**Table 1:** Conditions that Increase Adenosine in the CNS

Manipulation	Reference
Hypoxia	Fowler 1989 [55] Dale & Frenguelli 2000 [34] Saransaari & Oja 2003 [172] Martín, Fernández, Perea, Pascual, Haydon, Araque & Ceña 2007 [123]
Ischemia	Fowler 1990 [56] Latini, Corsi, Pedata & Pepeu 1995 [108] Frenguelli, Llaudet & Dale 2003 [63] Parkinson, Xiong & Zamzow 2005 [155] Frenguelli, Wigmore, Llaudet & Dale 2007 [64]
NMDA receptor activation	Manzoni, Manabe & Nicoll 1994 [119] Semba & White 1997 [176] Melani, Corsi, Giménez-Llort, Martínez, Ogren, Pedata & Ferré 1999 [130] Brambilla, Chapman & Greene 2005 [21]
H_2_O_2_	Masino, Mesches, Bickford & Dunwiddie 1999 [129] Saransaari & Oja 2003 [172]
Hypoglycemia or impaired glycolysis	Fowler 1993 [57] Zhu & Krnjević 1993 [201] Calabresi, Centonze, Pisani & Bernardi 1997 [22] Zhao, Tekkök & Krnjević [200] Minor, Rowe, Soames Job, Ferguson [131]
Increased temperature	Gabriel, Klussman & Igelmund 1998 [67] Masino & Dunwiddie 1999 [124]
Hypercapnia/acidification	Dulla, Dobelis, Pearson, Frenguelli, Staley & Masino 2005 [42] Gourine, Llaudet, Dale & Spyer 2005 [77] Otsuguro, Yamaji, Ban, Ohta & Ito 2006 [146]
Depolarization	Pedata, Pazzagli, Tilli & Pepeu 1990 [157] Latini, Corsi, Pedata & Pepeu 1995 [108]
Metabolic poisons	Doolette1997 [40] Zhu & Krnjević 1997 [202] Saransaari & Oja 2003 [172]
Astrocyte activation	Zhang, Wang, Ye, Ge, Chen, Jiang, Wu, Poo & Duan 2003 [199] Parkinson & Xiong 2004 [154] Pascual, Casper, Kubera, Zhang, Revilla-Sanchez, Sul, Takano, Moss, McCarthy & Haydon 2005 [156]
Seizures	Whitcomb, Lupica, Rosen & Berman 1990 [193] During & Spencer 1993 [46] Berman, Fredholm, Aden & O’Connor 2000 [11] Kaku, Jiang, Hada, Morimoto & Hayashi 2001 [93] Etherington, Patterson, Meechan, Boison, Irving, Dale & Frenguelli 2008 [52]
Intense exercise	Dworak, Diel, Voss, Hollman & Strüder 2007 [47]
Sleep deprivation	Porkka-Heiskanen, Strecker, Thakkar, Bjorkum, Greene & McCarley 1997 [162] Porkka-Heiskanen, Strecker & McCarley 2000 [161] Kalinchuk, McCarley, Stenberg, Porkka-Heiskanen & Basheer 2008 [94] Murillo-Rodriguez, Liu, Blanco-Centurion & Shiromani, 2008 [133]

An overview of both physiological and pathological conditions of altered metabolism and cellular activity that can increase extracellular adenosine. Due to the rapid dephosphorylation of extracellular ATP to adenosine, increased extracellular ATP yields a net increase in adenosine. This table is not meant to be exhaustive of the literature, but to highlight the ubiquitous and rapid nature of the adenosine response and thus its broad and dynamic influence in the nervous system.

**Table 2:** Influence of Ketone Metabolism on Cellular Energy

	Energetic Molecules	Expression of Mitochondrial Genes or Proteins	Mitochondria	Respiration
Brain	*Increased ATP [37, 99, 127, 134, 143]* *Increased phosphocreatine [20, 151]*	*Upregulated ATP synthase [20, 141]* *Upregulated uncoupling protein [182]* Unchanged succinate dehydrogenase [167]	*Increased number [20, 143]* *Increased *or reduced number (region-dependent) [6]	*Increased respiration [182]*
Peripheral tissues	Unchanged ATP production [158]	Unchanged citrate synthase [158] Unchanged succinate dehydrogenase [137, 167]	*Moderately increased size [48]* Unchanged [6]	

Evidence for changes in cellular energy in brain and peripheral tissues after ketogenic metabolism in vivo or in vitro. An increase or upregulation is indicated by italics throughout. “Expression of genes or proteins” includes mRNA expression and protein expression via immunochemical or activity-based assays; here we include both cell and mitochondriarelated genes/proteins. Peripheral tissues include skeletal muscle and liver.
