# ERRATUM 1

**DOI:** 10.2174/157015910790909494

**Published:** 2010-03

This is with reference to the article entitled, “**Adenosine, Ketogenic Diet and Epilepsy: The Emerging Therapeutic Relationship Between Metabolism and Brain Activity**”, by, S.A. Masino, M. Kawamura Jr., C.D. Wasser, L.T. Pomeroy and D.N. Ruskin published in *Current Neuropharmacology* Journal, 257-268 September **2009,** Vol. *7*, No. *3*, pp. 257-268 .

Due to an oversight, author middle name was cited as Caleb A. Wasser and it should read as C.D. Wasser.

